# Effect of CIP2A and its mechanism of action in the malignant biological behavior of colorectal cancer

**DOI:** 10.1186/s12964-020-00545-6

**Published:** 2020-04-22

**Authors:** Wei Chen, Jing-Lin Liang, Kai Zhou, Qing-Li Zeng, Jun-Wen Ye, Mei-Jin Huang

**Affiliations:** 1 Department of Colorectal Surgery, The Six Affiliated Hospital, Sun Yat-sen University, Guangzhou, 510655 China; 2 Guangdong Provincial Key laboratory of Colorectal and Pelvic Floor Disease, The Sixth Affiliated Hospital of Sun Yat-sen University, Guangzhou, 510655 China; 3 Guangdong Research Institute of Gastroenterology, The Sixth Affiliated Hospital of Sun Yat-sen University, Guangzhou, 510655 China; 4 Jiangxi Provincial People’s Hospital, Nanchang, 330006 Jiangxi China; 5 The 334 Hospital Affiliated of Nanchang University, Nanchang, 330024 Jiangxi China

**Keywords:** Colorectal cancer, CIP2A, Cell proliferation, Serum, 10,058-F4

## Abstract

**Background:**

Increasing evidence has revealed a close correlation between cancerous inhibitor of protein phosphatase 2A (CIP2A) and cancer progression. CIP2A has been shown to participate in diverse biological processes, such as development, tumorigenic transformation and chemoresistance. However, the functions of CIP2A in colorectal cancer (CRC) and its underlying mechanisms of action are not yet completely understood. The purpose of this study was to explore its clinical significance, function and relevant pathways in CRC.

**Methods:**

Quantitative RT-PCR (qRT-PCR), immunohistochemistry (IHC), western blotting and enzyme-linked immunosorbent assay (ELISA) were used to identify the expression of CIP2A in CRC tissues, sera and CRC cell lines. The association between the expressions of CIP2A and patient survival was analyzed using the Kaplan-Meier curves. Additionally, the functional role of CIP2A in the cell lines was identified through small interfering RNA (siRNA)-mediated depletion of the protein followed by analyses of proliferation and xenograft growth in vivo using short hairpin (sh) RNAs. Effects of the C-myc inhibitor 10,058-F4 on the expressions of C-myc, and CIP2A in CRC cell lines and its potential mechanisms of action were investigated. Finally, the potential molecular pathways associated with CIP2A were screened using the phosphokinase array and identified through western blotting.

**Results:**

CIP2A mRNA and protein levels were upregulated in CRC tissues compared to those of the corresponding normal tissues. It can be used as an independent prognostic indicator to determine overall survival (OS) and disease-free survival (DFS). Depletion of CIP2A substantially suppressed the growth of CRC cells and colony formation in vitro, and inhibited the growth of xenograft tumors in vivo. Additionally, the levels of CIP2A in the sera of patients with CRC were higher than those of the control subjects. Multivariate analyses revealed that the levels of CIP2A in the sera were not independent prognostic indicators in patients with CRC. Moreover, 10,058-F4 could effectively inhibit the growth of CRC cells in vitro, which could be correlated with an inhibition in the expressions of C-myc, CIP2A and its downstream regulatory anti-apoptotic proteins. Furthermore, the Human Phosphokinase Antibody Array was used to gain insights into the CIP2A-dependent intermediary signaling pathways. The results revealed that several signaling pathways were affected and the protein levels of p-p53 (S392), p-STAT5a (Y694), Cyclin D1, p-ERK1/2 and p-AKT (T308) had decreased in CIP2A-shRNA group based on the results of the western blot analysis.

**Conclusions:**

CIP2A could promote the development of CRC cells and predict poor prognosis in patients with CRC, suggesting that it may serve as a potential prognostic marker and therapeutic target against CRC.

Video Abstract

**Graphical abstract:**

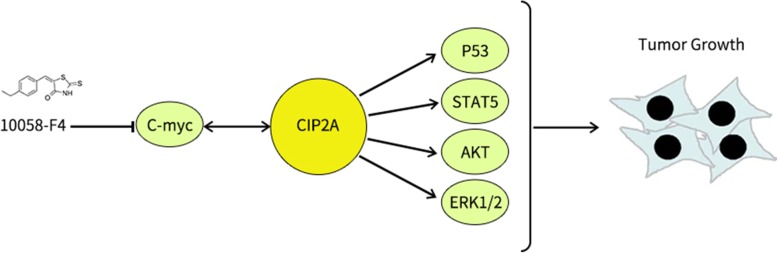

## Background

Colorectal cancer (CRC) is one of the most frequently occurring malignant tumors of the digestive tract and its prevalence has risen rapidly in the recent years [1]. Although the increased prevalence of chemoradiation therapy along with surgery has improved the regional and local control of CRC in patients with a locoregionally advanced level of the disease, the overall survival (OS) rate is still unsatisfactory [2]. Moreover, few markers besides the TNM stage have been used as diagnostic criteria. Therefore, a better understanding of the molecular mechanisms associated with CRC is essential for improving its clinical outcome.

CIP2A (cancerous inhibitor of protein phosphatase 2A), which has been identified as a PP2A-interacting protein with endogenous growth, prevents the proteolytic degradation of C-myc by preventing PP2A-mediated dephosphorylation at serine 62 [3]. MYC and CIP2A are regulated through a positive feedback loop, which promotes the expression of both proteins [4]. Currently, it has been determined that CIP2A is overexpressed in the sera and cells of patients with different types of cancers, such as prostate, breast, and hepatocellular carcinoma (HCC) [5–8]. However, its application as an indicator, which can be detected in the sera of patients with CRC has not yet been reported. In addition, as a small molecular targeting inhibitor of C-myc which is associated with the progression of cancer, 10,058-F4 has been recognized as a potent molecular targeting drug for the treatment of a variety of tumors [9–12]. It is still unknown whether 10,058-F4, which inhibits the interaction of C-myc/MAX, can indirectly suppress the expression of CIP2A by disrupting the interaction of CIP2A/C-myc in patients with CRC.

Based on the results of current studies, we examined the expression of CIP2A in CRC cell lines and samples of patients with CRC, and identified the signaling pathways associated with it using a xenograft tumor model and phosphokinase array in CRC cell lines. Additionally, the effects of 10,058-F4 on the biological behavior of CRC cells and the expression of CIP2A were investigated. The results of this study could help elucidate the signaling pathways associated with the molecular mechanisms of CIP2A, rendering it as a promising target protein for the treatment of CRC.

## Materials and methods

### Cell culture and treatment

Some kinds of CRC cell lines (SW620, HCT116, LoVo, HT29 and DLD1) were obtained from the Culture Collection of the Chinese Academy of Science (Shanghai, China), cultured in RPMI 1640 medium or DMEM (Invitrogen) supplemented with 10% FBS, and incubated under conditions of 5% CO_2_ at 37 °C.

### Patients and specimens

A total of 390 paraffin-embedded samples of CRC tissues were obtained from the Department of Pathology, the Sixth Affiliated Hospital of Sun Yat-sen University (Guangzhou, P. R.China) between January 2000 and November 2006. Additionally, some specimens were collected for conducting quantitative RT-PCR (qRT-PCR) (26 pairs of fresh CRC and normal adjacent tissues) and the enzyme-linked immunosorbent assay (ELISA) (sera from 63 patients with CRC before surgery and 16 normal individuals). The study was approved by the Institute Research Medical Ethics Committee of Sun Yat-Sen University. Surgical staging was performed based on the criteria proposed by the International Union Against Cancer (UICC).

### Tissue microarray (TMA) and immunohistochemistry (IHC)

The TMA was constructed as described previously [13]. According to the manufacturer’s protocol, the Envision System with diaminobenzidine (DAKO Cytomation, Glostrup, Denmark) was used for IHC staining.

The TMA tissue blocks were cut into sections of 5-μm sections and incubated with the anti-CIP2A (Santa Cruz, CA) antibody. The staining index (values 0–12) was calculated based on its intensity (strong, 3; moderate, 2; weak, 1; or negative, 0 scores) and the proportion of positively stained CIP2A was determined and scored (< 25%, 1; 25–50%, 2; > 50–75%, 3; ≥75%, 4 scores). A staining index of > 6 was considered to indicate high expression. The samples were assessed independently by two pathologists.

### qRT-PCR

Total RNA from the samples of CRC and cell lines was isolated using TRIzol (Invitrogen) according to the manufacturer’s instructions. The first strand of complementary DNA (cDNA) was reverse transcribed using the SuperScript First-Strand cDNA System (Invitrogen) and amplified using PCR. The mRNA of β-actin was used as the reference gene and the experiment was performed in triplicates. The primer pairs used for qRT-PCR were as follows:

CIP2A, For: 5′-GAACAGATAAGAAAAGAGTTGAGCATT-3′,

Rev.: 5′-CGACCTTCTAATTGTGCCTTTT-3′.

### RNA interference, MTT assays and colony formation

According to the manufacturer’s instructions, the siControl and siRNA for CIP2A obtained from Dharmacon (Chicago, IL, USA) were transfected into cells using DharmaFECT 1 (ThermoFisher Scientific). The efficacy of siRNA at the mRNA and protein levels was evaluated 48 h after transfection.

For the MTT assay, 3 × 10^3^ cells of DLD1 and HT29 treated with siRNA were seeded onto 96-well plates and cultured for 24 h. Then, 20 μl of a solution of 5 mg/ml MTT (final concentration, 0.5 mg/ml) was added to each well and incubated for 4 h at 37 °C. Finally, the absorbance was measured at 490 nm using a microplate reader.

For the colony formation assay, DLD1 and HT29 cells transfected with siRNA were plated onto cell culture dishes, with a diameter of 6 cm, at a density of 500 cells/well and maintained for 12 d. Visible colonies (containing > 50 cells) were identified through Giemsa staining.

### Elisa

The serum antibody against CIP2A was evaluated using ELISA [14]. Briefly, 96-well microtiter plates were coated for 24 h with 2 μg/ml of CIP2A diluted in phosphate buffered saline (PBS) and incubated for 24 h at 4 °C. The plate was sealed using gelatin and the solution was allowed to stand at room temperature for 2 h. The antigen-coated wells were incubated with human serum diluted to 1:200 with PBS and incubated at room temperature for 2 h. The substrate 2,2′*-*azino-bis (3-ethylbenzothiazoline-6-sulfonic acid) (ABTS) (Invitrogen) and anti-human IgG-HRP (Invitrogen, NY) were used for detection reagent. The optical density (OD) was measured at a wavelength of 405 nm and a standard curve was obtained using the provided standards and used to identify the quantity of CIP2A in each serum sample. The experiment was repeated thrice and the average values were noted.

### CIP2A shRNA depletion in CRC cell lines

The Open Biosystems Expression Arrest GIPZ Lentiviral shRNAmir system (cat. no. RHS4430–98912354) was used to construct DLD1 and HT29 cells with shRNA depletion according to the manufacturer’s protocol. The packaging vectors (pCMV∆R8.2 and pHCMV-G) were transfected at concentrations of 5 μg into 5 × 10^5^ 293FT cells together with 10 μg of pGIPZ-CIP2A shRNA. After 48 and 72 h, the supernatants containing the viruses were collected and filtered. DLD1 and HT29 cells were infected with the CIP2A lentivirus and the infected cells were selected using 2.5 μg/μl of puromycin 24 h later (Sigma-Aldrich, St. Louis, MO).

### 10,058-F4 treatment, viability assay, analysis of cell cycle and apoptosis

DLD1 and HT29 cells in the 96-well microplates (6000 cells/well) were treated with different concentrations of 10,058-F4 (Sigma, St. Louis, MO, USA) diluted in dimethylsulfoxide (DMSO), and incubated for 24, 48, and 72 h. Then, cells from each well were solubilized using 150 μl DMSO after incubation with the MTT reagent for 4 h, and absorbance of the samples was measured using a microplate reader at a wavelength of 570 nm.

DLD1 and HT29 cells (3 × 10^5^ cells/well) were treated with different concentrations of 10,058-F4 (25 μmol/L, 50 μmol/L and 100 μmol/L) in 6-well plates for 24 h. The treated cells were harvested, fixed overnight in cold 70% ethanol at 4 °C, washed twice with chilled PBS, incubated with 100 μl RNAase (final concentration, 20 μg/ml) at 37 °C for 30 min, and stained using 400 μl of propidium iodide (PI; final concentration, 50 μg/ml) for 30 min. The labeled cells were analyzed by flow cytometry using the ModFit Software (Verity Software House Inc., USA). Additionally, the fluorescein isothiocyanate-labeled AnnexinV/propidium iodide Apoptosis Detection Kit was used to analyze the apoptosis of DLD1 and HT29 cells treated with 10,058-F4 according to the manufacturer’s instructions. A FACScan flow cytometer with The Cell Quest software was used for data acquisition and analysis. The following proteins were evaluated by western blotting: CIP2A (Santa Cruz, CA), C-myc (Cell Signaling, USA), Bcl-2 (Cell Signaling, USA), Bax (Cell Signaling, USA), Cyclin D1 (Cell Signaling, USA), p21 (Cell Signaling, USA), p27 (Cell Signaling, USA) and β-actin (Santa Cruz).

### Effects of CIP2A depletion on tumor growth in a xenograft tumor model

All of the 4-w-old male BALB/c nude mice used in this experiment were purchased from the Medical Experimental Animal Center of Guangdong Province (Guangzhou, China). The procedures performed on the animals were approved by the institutional ethical guidelines provided by the Animal Ethics Committee of the Sun Yat-sen University. DLD1 and HT29 cells stably expressing shCIP2A or scrambled control shRNA were injected into the dorsal flank of each mouse at concentrations of 1 × 10^6^ cells/injection site. The tumor volume was measured as follows: (length × width^2^)/2. The development of tumors was examined every 3 d and the mice were sacrificed after 4 w. The excised tumors were fixed and expressions of CD34 (Dako, USA) and VEGF (Santa Cruz, CA) were determined through IHC.

### Phosphokinase array

To identify the relative levels of phosphorylation at the 46 kinase phosphorylation sites, the Proteome Profiler Human PhosphoKinase Array Kit (ARY003B, R&D Systems) was used in the DLD1 and HT29 cells transfected with the control and CIP2A-specific shRNAs according to the manufacturer’s instructions. The resulting spots were identified and images were quantified using the Image lab software (Bio-Rad) and Microsoft Excel software. The proteins were evaluated through western blotting, which were identified as follows: p53 (Abcam), p-p53 (S392) (Abcam), STAT5a (Cell Signaling, USA), p-STAT5a (Y694) (Cell Signaling, USA), Cyclin D1 (Cell Signaling, USA), Bax (Cell Signaling, USA), p21 (Cell Signaling, USA), p-AKT (T308) (Cell Signaling, USA), AKT (Cell Signaling, USA), p-ERK1/2 (Cell Signaling, USA), ERK1/2 (Cell Signaling, USA), and β-actin (Santa Cruz).

### Statistical analysis

Each experiment was performed at least in triplicates. The data are presented as the mean ± SD. The relationship between the expression of CIP2A and clinicopathologic variables of patients with CRC was analyzed through the X^2^ test. The Kaplan–Meier method and log-rank test were used to assess survival rates, and hazard ratios (HRs) were calculated using the Cox proportional hazard regression models. Independent prognostic factors were tested using the multivariate Cox regression analysis. All statistical analyses were performed using the SPSS 11.0 software and *P* values of < 0.05 were considered to be statistically significant.

## Results

### Expression of CIP2A in clinical tissue specimens and cell lines

**Fig. 1:**
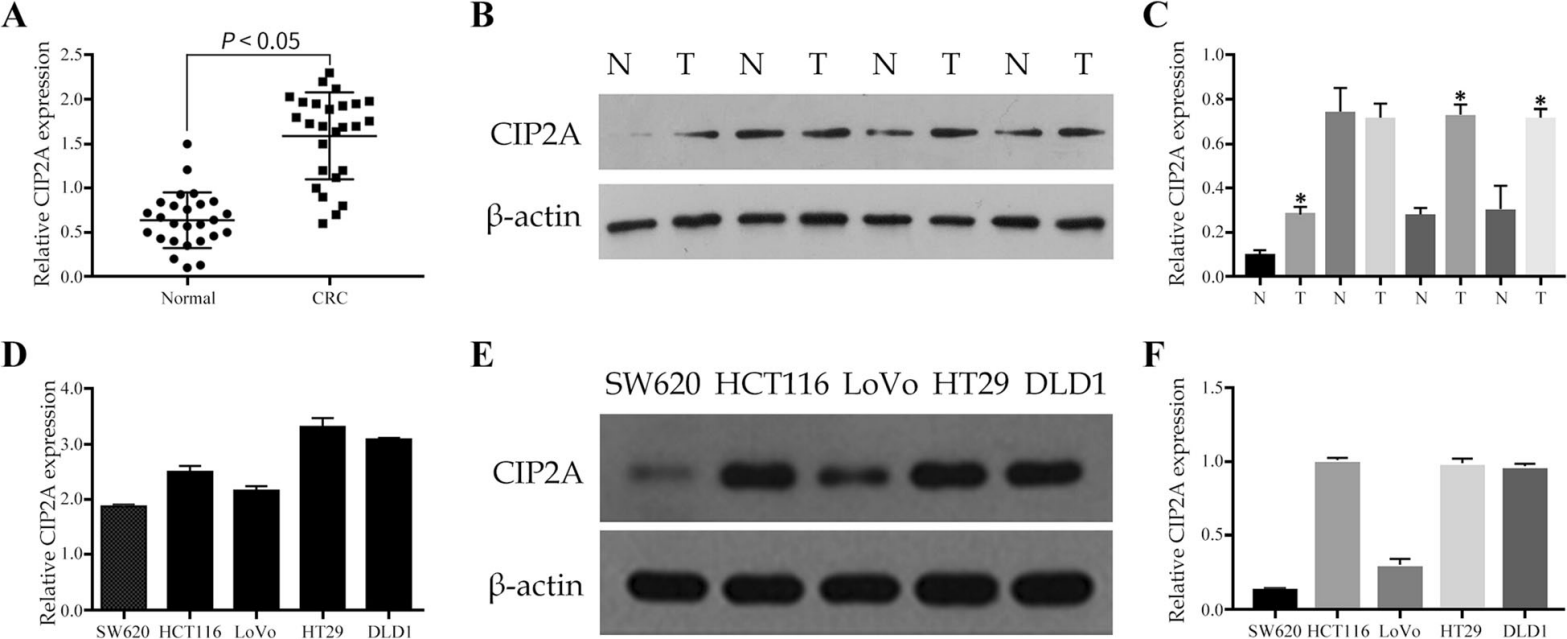
Expression levels of CIP2A in CRC cell lines and clinical samples. **a**, **b** Expression levels of CIP2A mRNA and protein in CRC tumor tissues (T) and normal tissues (N); (**d**, **e**) Expression levels of CIP2A mRNA and protein in CRC cell lines. (**c**, **f**) Statistical plots showing the relative proteins expression in CRC tissues sample and cell lines, respectively. *P < 0.05 compared to the control using Student’s *t*-test

The qRT-PCR was employed to identify the expression of CIP2A mRNA in the clinical tissue samples. Of the 26 paired specimens collected from the patients with CRC, the frequency of CIP2A expression was found to be significantly elevated in the CRC tissues (21/26, 80.7%) compared to the corresponding normal tissues (4/26, 15.3%; *P* < 0.05; Fig. 1a). Consistent with this result, expression of the CIP2A protein was also found to be significantly higher in the CRC tissues than in the corresponding normal tissues (Fig. 1b, c). Additionally, we determined the levels of CIP2A mRNA in various CRC cell lines. As shown in Fig. 1d, the expression of CIP2A mRNA was relatively higher in the CRC cell lines HCT116, HT29, and DLD1. Western blot analysis using the anti-CIP2A antibody revealed a single band at approximately 90 kDa. The CIP2A protein was expressed in all five CRC cell lines with evident differential expressions and was relatively higher in the HCT116, HT29, and DLD1 (Fig. 1e, f).

### Correlation of the expression of the CIP2A protein with the clinicopathologic parameters and survival analysis

**Fig. 2:**
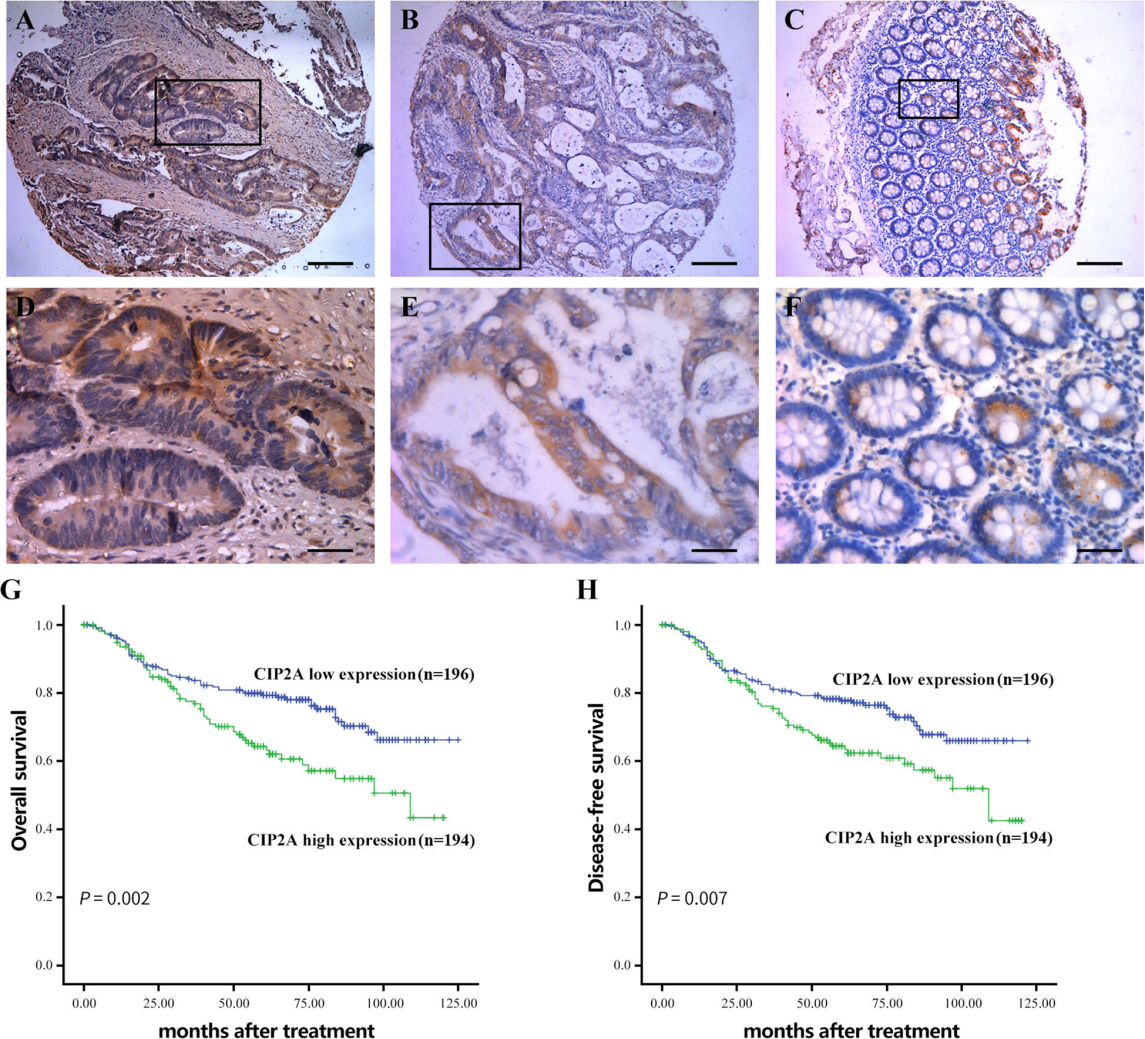
Expression levels of CIP2A and survival of patients with CRC. **a**, **d** Strong staining: brown (A: 40×; D: 200×); (**b**, **e**) Weak staining: light yellow (B: 40×; E: 200×); (**c**, **f**) Negative staining (C:40×; F:200×); The scale bars are 200 μm (**a**, **b**, **c**) and 50 μm (**d**, **e**, **f**), respectively; (**g**-**h**) Patients with high CIP2A expression (*n* = 194) had poorer OS and DFS than patients with low CIP2A expression (*n* = 196)

IHC analysis was carried out to determine the expression of CIP2A on the microarray of CRC and the corresponding normal tissues. We observed that CIP2A was not expressed in the adjacent non-cancerous tissues (Fig. 2c, f). Contrarily, the expression of CIP2A was high in the CRC tissues (Fig. 2a, b, d, and e). We further analyzed the correlation between the expression of CIP2A and the clinicopathologic features of CRC. As summarized in Table 1, the expression of CIP2A was significantly associated with the stage of TNM (*P* = 0.010) and levels of preoperative CEA (*P* = 0.011). No significant correlation was observed between the expression of CIP2A and the gender, age, location, T stage, and N stage of patients (Table 1). Additionally, the Kaplan–Meier survival analysis revealed that the patients whose localized CRC highexpressed CIP2A had a significantly lower 5-year DFS and OS than patients with low CIP2A expression in their tumors (Fig. 2g, h). Furthermore, based on the univariate and multivariate analyses, the CIP2A, liver metastasis, TNM stage and preoperative CEA level were statistically significantly associated with DFS and OS (all *P* < 0.05, Tables 2, 3).

**Table 1:** Correlation between CIP2A expression and clinicopathologic characteristics of patients with CRC (*n* = 390)

Characteristics	Cases	CIP2A protein		***P***
		Low expression	High expression	
**Gender**				0.091
Female	176	97	79	
Male	214	136	78	
**Age**				0.838
≥ 65	164	97	67	
< 65	226	136	90	
**Location**				0.304
Colon	200	126	74	
Rectum	190	110	80	
**T stage**				0.395
T1-T2	106	67	39	
T3-T4	284	166	118	
**N stage**				0.253
N0	237	144	93	
N1-N2	153	84	69	
**M stage**				0.869
M0	344	205	139	
M1	46	28	18	
**TNM stage**				0.010
I-II	227	148	79	
III-IV	163	85	78	
**Histological grade**				0.357
Well	47	31	16	
Moderately	309	179	130	
Poorly	34	23	11	
**CEA**				0.011
≥ 5	161	74	87	
< 5	229	135	94	
**CA19–9**				0.057
≥ 37	103	48	55	
< 37	287	165	122	
**Family history**				0.291
No	375	224	151	
Yes	15	11	4	
**Liver metastasis**				0.291
No	368	220	148	
Yes	22	13	9

**Table 2:** Univariate and multivariate analyses of prognostic factors for 5-year DFS of patients with CRC

Variable	Univariate analysis		Multivariate analysis		***P***
	5 year-DFS	***P***	HR	CL (95%)	
**Gender**		0.190			NS
Female	72.0				
Male	74.4				
**Age**		0.989			NS
≥ 65	75.0				
< 65	70.6				
**Location**		0.967			NS
Colon	73.0				
Rectum	72.8				
**T stage**		0.011			NS
T1-T2	79.2				
T3-T4	69.7				
**N stage**		0.002			NS
N0	80.7				
N1-N2	61.7				
**M stage**		0.033			NS
M0	73.7				
M1	61.6				
**TNM stage**		0.000			0.010
I-II	77.7		1		
III-IV	64.7		1.114	0.877–1.414	
**Histological grade**		0.450			NS
Well	77.5				
Moderately	72.0				
Poorly	69.0				
**CEA**		0.019			0.000
≥ 5	68.0		1		
< 5	80.0		0.422	0.271–0.657	
**CA19–9**		0.164			NS
≥ 37	70.0				
< 37	79.9				
**Family history**		0.653			NS
No	73.2				
Yes	69.2				
**Liver metastasis**		0.000			0.000
No	76.0		1		
Yes	13.6		11.129	6.382–6.312	
**CIP2A**		0.007			0.042
Low expression	77.7		1		
High expression	63.3		1.478	1.014–2.155

**Table 3:** Univariate and multivariate analyses of prognostic factors for 5-year OS of patients with CRC

Variable	Univariate analysis		Multivariate analysis		***P***
	5 year-OS	***P***	HR	CL (95%)	
**Gender**		0.551			NS
Female	70.6				
Male	75.2				
**Age**		0.856			NS
≥ 65	74.6				
< 65	72.8				
**Location**		0.644			NS
Colon	74.7				
Rectum	73.5				
**T stage**		0.007			NS
T1-T2	82.4				
T3-T4	68.1				
**N stage**		0.013			NS
N0	78.1				
N1-N2	63.6				
**M stage**		0.041			NS
M0	74.4				
M1	62.1				
**TNM stage**		0.001			0.030
I-II	79.0		1		
III-IV	65.6		1.263	1.023–1.559	
**Histological grade**		0.561			NS
Well	81.9				
Moderately	72.2				
Poorly	75.0				
**CEA**		0.030			0.000
≥ 5	69.6		1		
< 5	80.3		0.410	0.256–0.646	
**CA19–9**		0.323			NS
≥ 37	71.7				
< 37	79.2				
**Family history**		0.619			NS
No	74.5				
Yes	69.2				
**Liver metastasis**		0.000			0.000
No	77.3		1		
Yes	12.8		10.718	6.011–19.111	
**CIP2A**		0.002			0.024
Low expression	79.3		1		
High expression	64.2		1.565	1.060–2.310	

### Knockdown of CIP2A in CRC cell lines inhibits cell proliferation

**Fig. 3:**
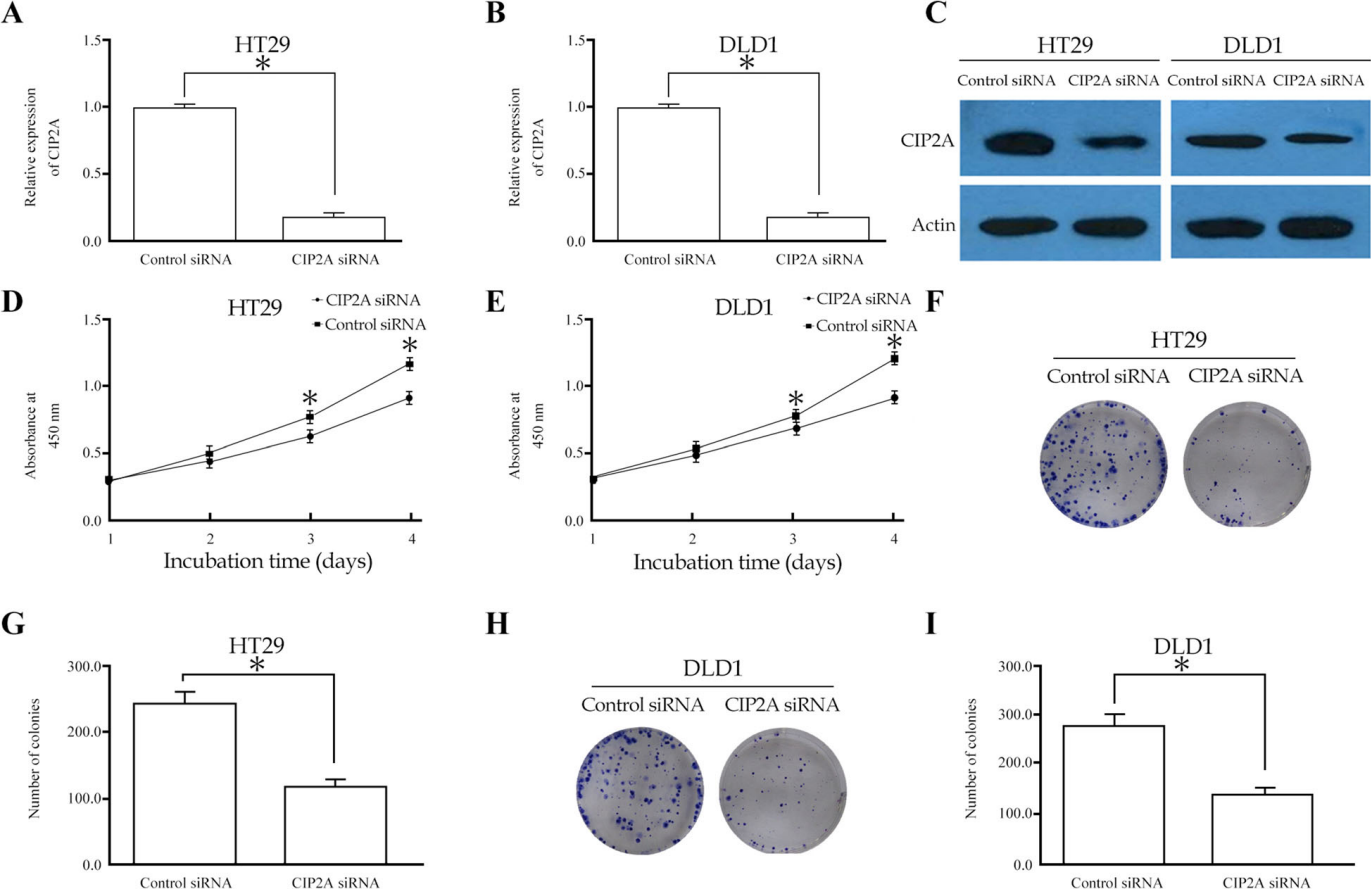
Effects of CIP2A depletion on its expression and proliferation of CRC cells in vitro. **a-c** Effects of CIP2A siRNA on CIP2A mRNA and protein expression in HT29 and DLD1 cells detected by RT-PCR (**a**, **b**) and western blot analysis (**c**); **d-i** Effects of CIP2A siRNA on the cell proliferation (**d**, **e**) and anchorage-independent growth (**f-i**) of HT29 and DLD1 cells (*n* = 3 for each cell line). *P < 0.05 compared to the control using Student’s *t*-test

To identify whether CIP2A inhibits the proliferation of CRC cells, we determined the levels of its expression in several CRC cell lines and found that it was relatively higher in HCT116, HT29, and DLD1 cells. The siRNA knockdown was carried out in DLD1 and HT29 cell lines. As shown in Fig. 3a-c, the expression of CIP2A mRNA and protein was clearly inhibited in the DLD1 and HT29 cells of the group infected with CIP2A siRNA. The rate of cell proliferation was determined using the MTT assay. A significant decrease in proliferation was observed in the DLD1 and HT29 cells treated with the CIP2A siRNA compared to those with negative siRNA (Fig. 3d, e). Consistent with the MTT results, the results of the colony formation assay also revealed that the knockdown of CIP2A in DLD1 and HT29 cells led to a significant decrease in the number of foci (*P* < 0.05, Fig. 3f-i).

### Effects of 10,058-F4 on the biological behavior of CRC cells and expression of CIP2A

**Fig. 4:**
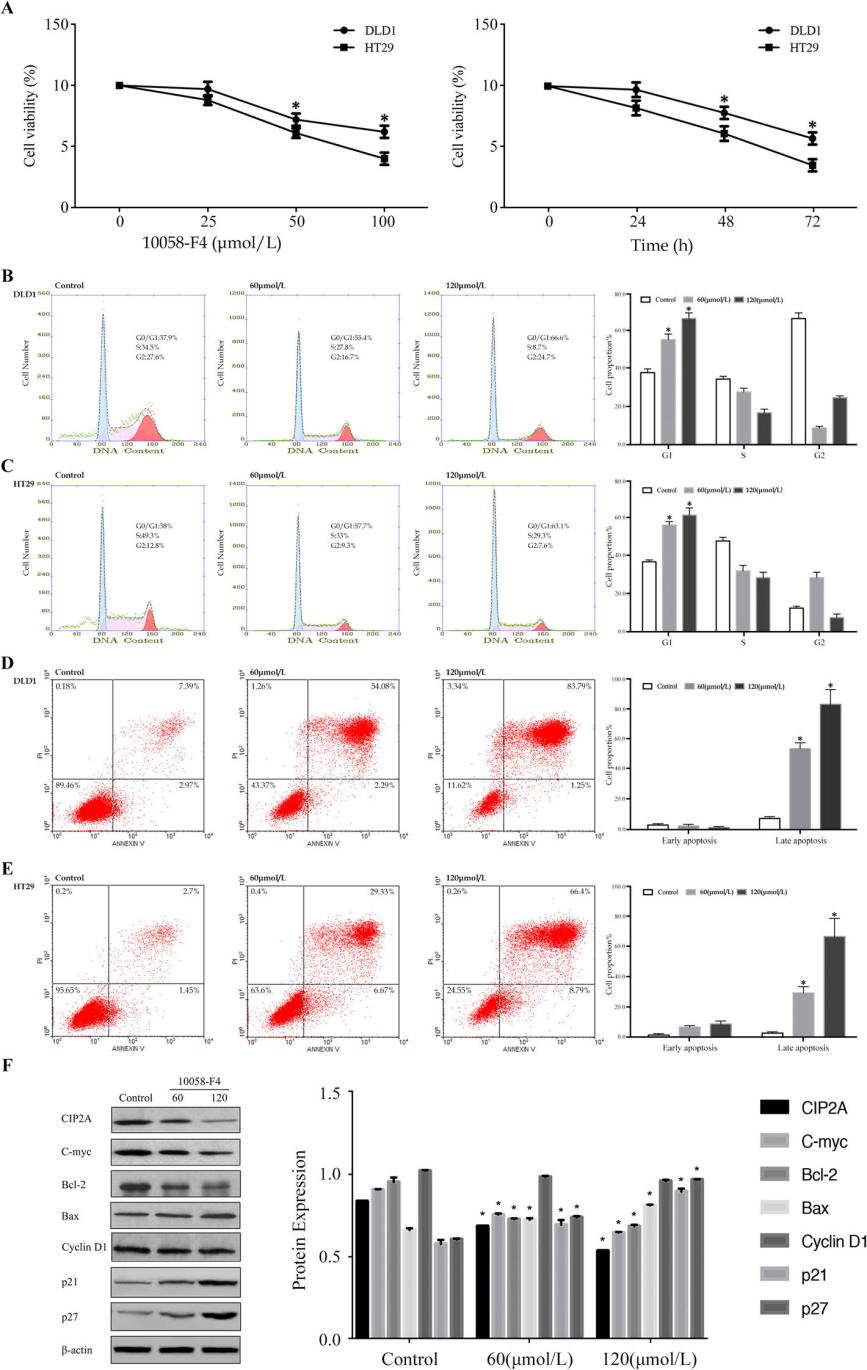
Effects of C-myc inhibitor 10,058-F4 on HT29 and DLD1 cells. **a** HT29 and DLD1 cells were treated with various concentrations of 10,058-F4 for 48 h and analyzed using the MTT assay. HT29 and DLD1 cells were treated with 50 μmol/l of 10,058-F4 for the indicated time and analyzed using the MTT assay; **b**, **c** HT29 and DLD1 cells were treated with 10,058-F4 for 48 h and analyzed using the flow cytometry for cell cycle analysis; **d**, **e** HT29 and DLD1 cells were treated with 10,058-F4 for 48 h and analyzed using the flow cytometry for the Annexin V-PI analysis; **f** Expression of the CIP2A, C-myc, Bcl-2, Bax, Cyclin D1, p21 and p27 proteins in CRC cells treated with 10,058-F4 was detected by western blotting. *P < 0.05 compared to the control using Student’s *t*-test

We first examined the effect of 10,058-F4 on CRC cells using the MTT assay. DLD1 and HT29 cells were found to be sensitive to 10,058-F4 in a time and concentration-dependent manner (Fig. 4a). The treatment with 10,058-F4 led to an increase in the accumulation of cells in the G1 phase in both cell lines (Fig. 4b, c), which was consistent with the suppression of growth observed during the MTT assays. Simultaneously, the percentages of DLD1 and HT29 cells undergoing late apoptosis had increased significantly in a dose-dependent manner (Fig. 4d, e).

To elucidate the mechanisms underlying cell cycle arrest and apoptosis, the expressions of associated proteins were examined. As is shown in Fig. 4f, we found that the expression of C-myc decreased significantly due to the effect of 10,058-F4 in a concentration-dependent manner. Interestingly, inhibition of C-myc led to a decrease in the levels of CIP2A. The expression levels of p21 and p27 increased and no significant change was observed in the expression level of Cyclin D1. Additionally, the activity of Bax, which plays a key role in apoptosis, had increased after treatment with 10,058-F4. However, an opposite trend was observed in the expression of the Bcl-2 protein.

### Expression of serum CIP2A and its correlation with prognosis of CRC in patients

**Fig. 5:**
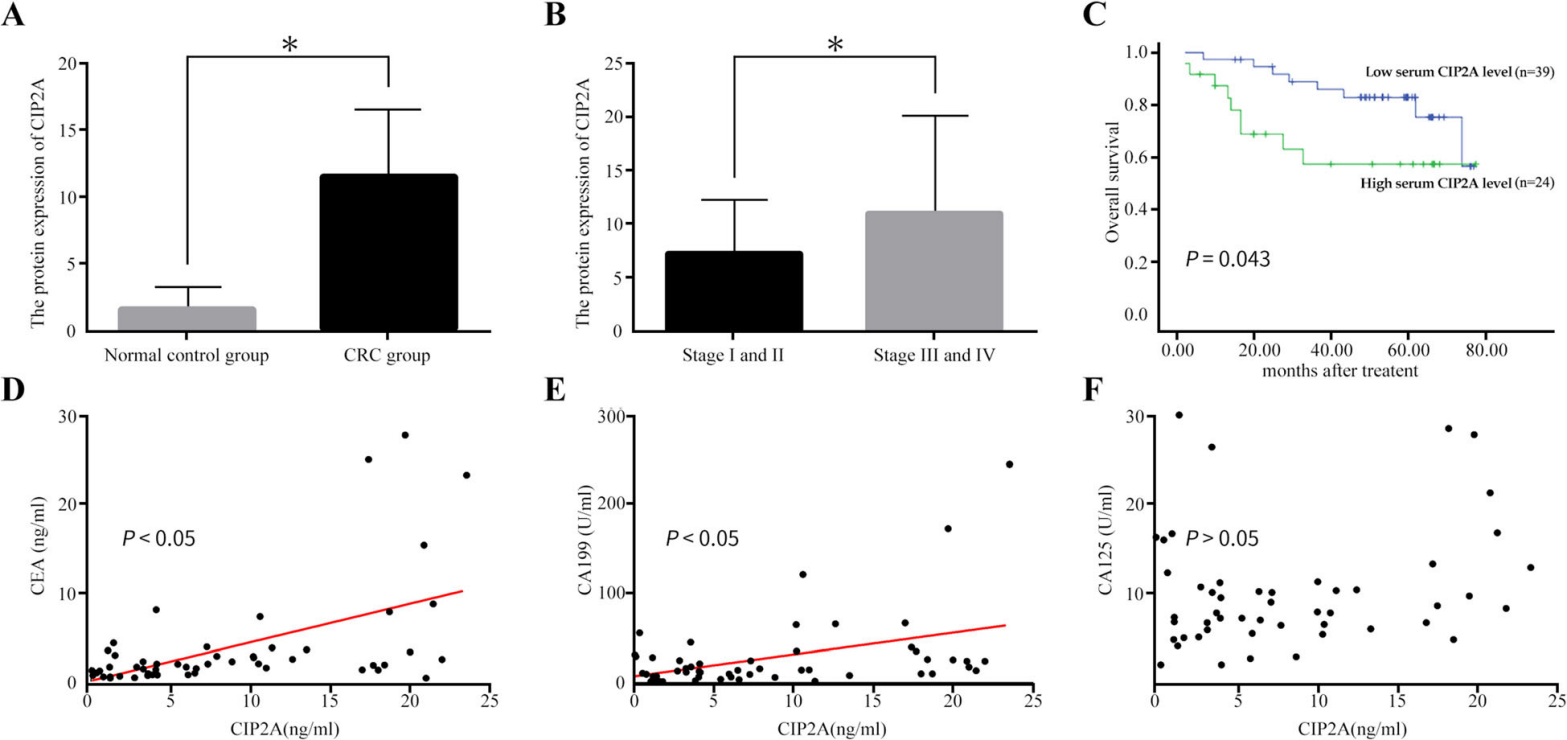
Evaluation of the expression of CIP2A in the sera of patients with CRC and its clinical significance. **a** The comparison of the level of CIP2A in CRC group and control; **b** The comparison of the level of CIP2A in different stage of CRC; **c** Patients with high serum CIP2A expression (*n* = 24) had poorer OS than patients with low serum CIP2A expression (*n* = 39); **d-f** The relationships between the expression of CIP2A in the sera of CRC patients and CEA (D), CA19–9 (**e**), and CA12–5 (**f**)

The concentrations of CIP2A had significantly increased in the sera of patients with CRC, compared to normal individuals (9.140 ± 0.937 vs. 1.659 ± 0.375 ng/mL, respectively; *P* = 0.000; Fig. 5a). The levels of CIP2A were found to be higher in the sera of patients with late stage CRC (III and IV) than in those with early stage (I and II) CRC (11.00 ± 1.62 ng/mL vs. 7.334 ± 0.875 ng/mL, respectively; *P* = 0.000; Fig. 5b). Additionally, the patients could be categorized into two groups, which included those with low or high levels of CIP2A in their sera, using the median expression level of all cases as the cut-off point. We analyzed that no significant correlation existed between the expression levels of CIP2A and the gender, age, location, T stage, and N stage of the patients (Table 4). Multivariate analyses revealed that only the T stage was an independent prognostic indicator of CRC in patients (*P* < 0.05, Table 5). The Kaplan–Meier analysis revealed that the survival trends in patients with low levels of serum CIP2A were favorable (*P* = 0.043, Fig. 5c). The mean survival rates for patients with low and high levels of serum CIP2A were 66.75 (95% CI, 60.272–73.230) and 51.43 (95% CI, 38.253–64.610) months, respectively. We observed a positive correlation between the levels of CIP2A and CEA (r = 0.353, *P* = 0.009, Fig. 5d), CIP2A and CA199 (*r* = 0.415, *P* = 0.002, Fig. 5e), but not CIP2A and CA125 (*r* = 0.074, *P* = 0.597, Fig. 5f).

**Table 4:** Correlation between serum CIP2A and clinicopathologic characteristics of patients with CRC (*n* = 63)

Characteristics	Cases	CIP2A protein		***P***
		Low expression	High expression	
**Gender**				0.960
Female	37	23	14	
Male	26	16	10	
**Age**				0.967
≥ 65	25	15	10	
< 65	38	23	15	
**Location**				0.163
Colon	17	7	10	
Rectum	46	28	18	
**T stage**				0.775
T1-T2	35	20	15	
T3-T4	28	17	11	
**N stage**				0.907
N0	40	22	18	
N1-N2	23	13	10	
**M stage**				0.407
M0	37	21	16	
M1	26	12	14	
**TNM stage**				0.509
I-II	37	23	14	
III-IV	26	14	12	
**Histological grade**				0.368
Well	26	17	9	
Poorly-Moderately	37	20	17	
**CEA**				0.002
≥ 5	10	2	8	
< 5	53	38	15	
**CA19–9**				0.014
≥ 37	14	4	10	
< 37	49	32	17

**Table 5:** Univariate and multivariate analyses of association of baseline prognostic factors with 5-year OS of patients with CRC

Variable	Univariate analysis		Multivariate analysis		***P***
	5 year-OS	***P***	HR	CL (95%)	
**Gender**		0.231			NS
Female	82.8				
Male	69.6				
**Age**		0.256			NS
≥ 65	61.3				
< 65	79.1				
**Location**		0.166			NS
Colon	60.2				
Rectum	73.7				
**T stage**		0.000			0.029
T1-T2	90.0		1		
T3-T4	49.8		8.905	1.256–63.182	
**N stage**		0.004			NS
N0	81.1		1		
N1-N2	50.0		6.225	0.454–85.377	
**M stage**		0.000			NS
M0	78.1		1		
M1	34.3		6.383	0.905–45.004	
**TNM stage**		0.001			NS
I-II	86.9		1		
III-IV	26.4		0.195	0.009–4.458	
**Histological grade**		0.124			NS
Well	90.9				
Moderately	72.7				
Poorly	48.5				
**CEA**		0.003			NS
≥ 5	34.3		1		
< 5	76.7		1.344	0.269–6.711	
**CA19–9**		0.117			NS
≥ 37	54.5				
< 37	67.1				
**CIP2A**		0.043			0.052
Low expression	75.4		1		
High expression	57.4		4.301	0.988–18.725	

### Effects of CIP2A depletion on tumor growth

**Fig. 6:**
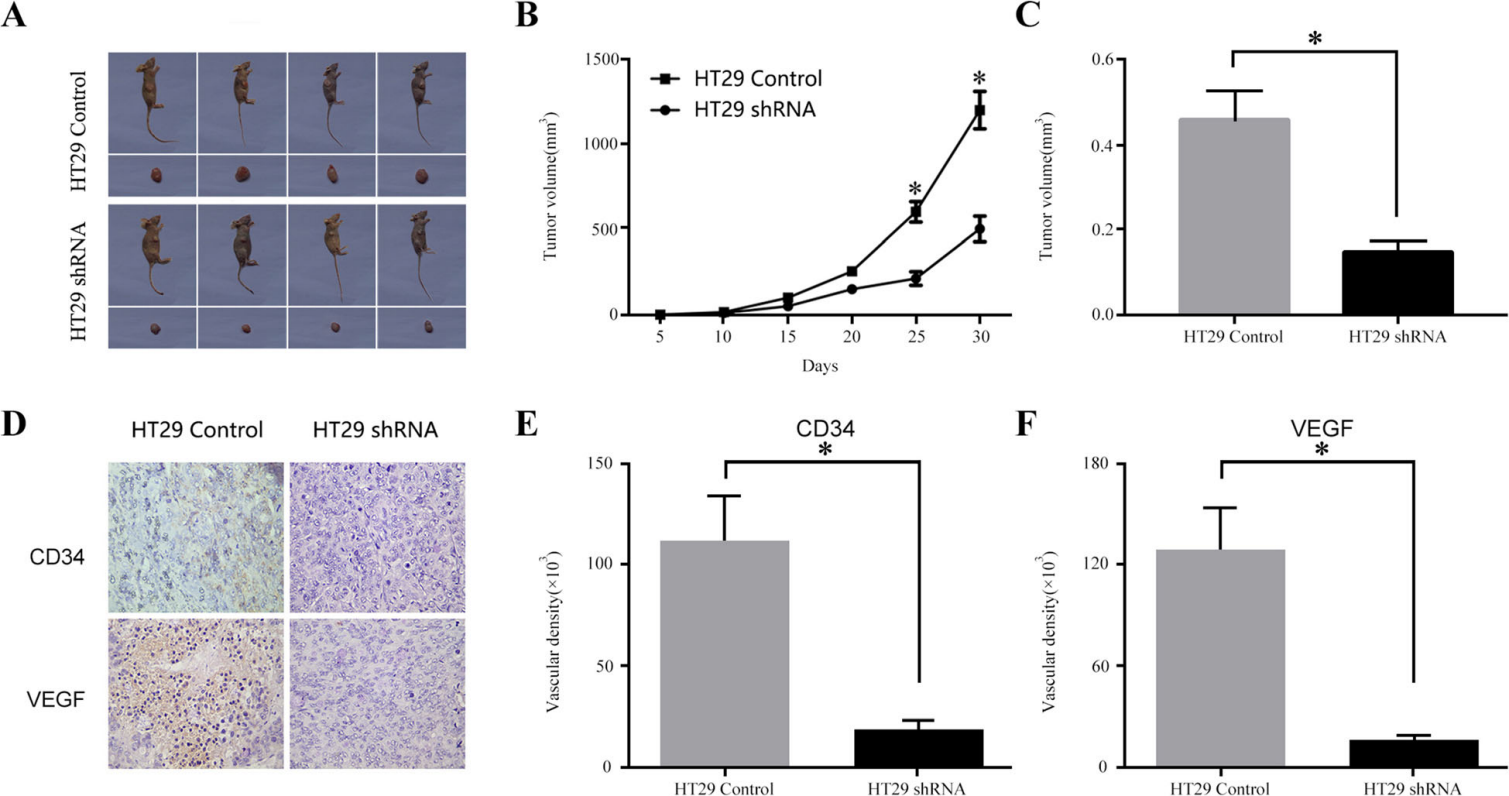
Effects of CIP2A depletion on the CRC xenograft tumor growth in vivo. **a** HT29 cells stably expressing shCIP2A or scrambled control were subcutaneously injected into nude mice. HT29 cells stably expressing shCIP2A had smaller tumors than scrambled controls after 4 w; **b** The growth curves of tumor volumes; **c** Tumor weights; **d** Application of IHC to detect the expressions of CD34 and VEGF in different groups of xenograft tumors (*n* = 10/group); **e**, **f** Statistical plot showing the relative protein expression of CD34 and VEGF in different groups (*n* = 10/group) of xenograft tumors, respectively. *P < 0.05 compared to the control using Student’s *t*-test

To detect whether CIP2A silencing had influenced the growth of tumors in vivo, the CIP2A-shRNA and control vectors were transfected into HT29 cells, which were then subcutaneously injected into the right flank of each nude mouse at concentrations of 1 × 10^6^ cells. We observed that the proliferation rate of the cells in the group with CIP2A depletion was lower and had resulted in the formation of substantially smaller tumors than those in the control groups. The average tumor volumes and weights in the CIP2A-shRNA group had also prominently decreased compared to those in the control group (Fig. 4a-c). Furthermore, IHC on the xenografts was carried out using CD34 and VEGF, which are markers for tumor angiogenesis. As shown in Fig. 6d–f, a strong activity for CD34 and VEGF was observed in the vessels of the CIP2A-control tumors. Therefore, we observed that these findings were consistent with the results of the cell proliferation assay, which indicated that CIP2A is involved in the progression of CRC.

### Determination of the protein phosphorylation patterns associated with CIP2A

**Fig. 7:**
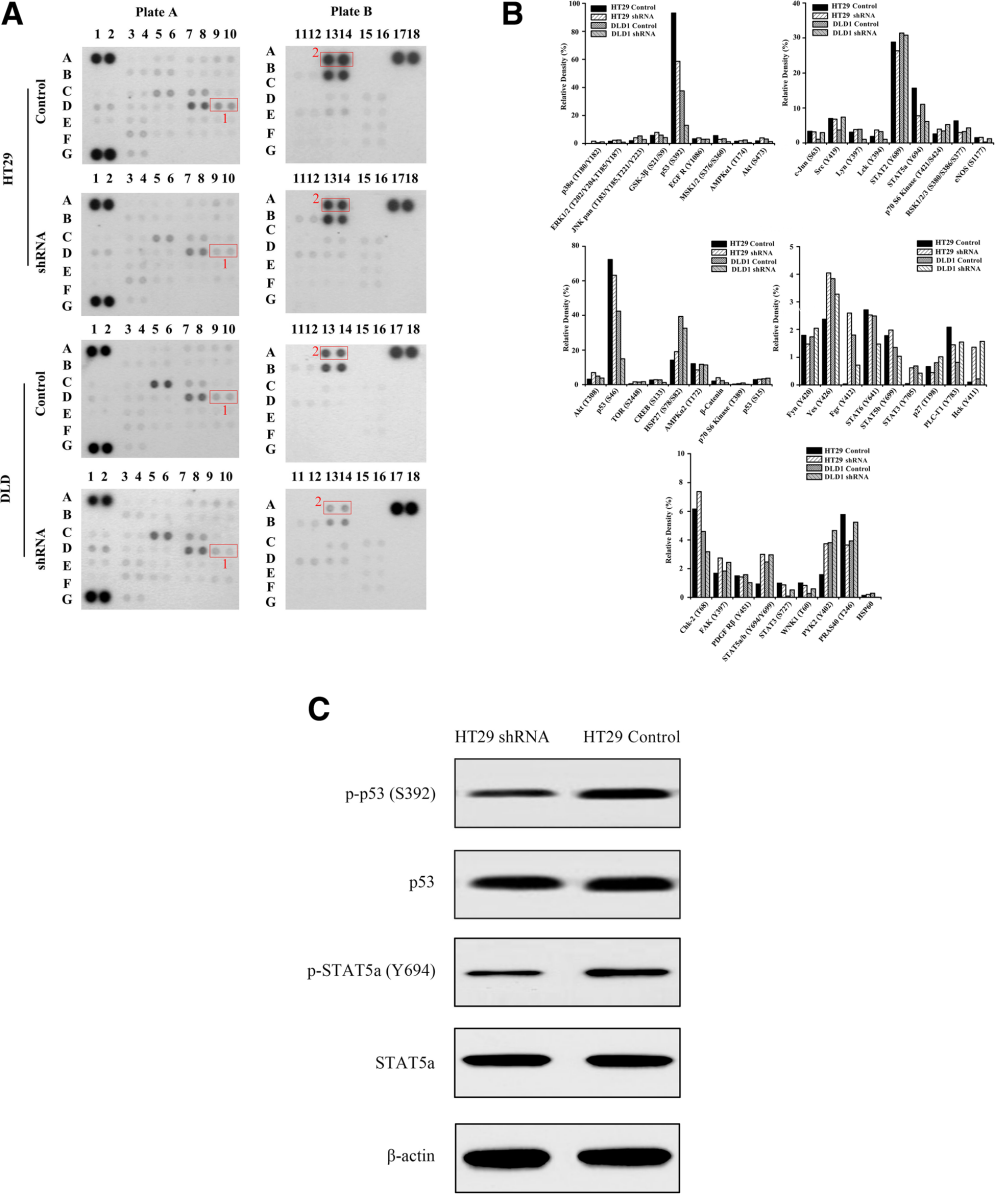
Illustration and representative image of the phosphokinase antibody array using lysates of CRC cells with scramble control and CIP2A-shRNA. **a** Detection of phosphorylated proteins in CRC cells with scramble control and CIP2A shRNA using the phosphokinase array; The 1 and 2 in the box represents STAT5a (Y694) and p53(S392) respectively; **b** Statistical plot showing the relative expressions of phosphorylated proteins (*n* = 2/group) associated with CIP2A; **c** The p-p53 (S392) and p-STAT5a (Y694) were identified by western blotting and each western analysis was repeated for three times or more

Currently, the signaling pathways associated with CIP2A in CRC, are not well known. Thus, the phosphorkinase array was set up. In Fig. 7a, b, we can observe that the number of phosphorylated proteins with a decreased expression were 17 and 26, of which, 8 and 22 types demonstrating a decrease in the phosphorylation levels over 20% were observed in HT29 and DLD with CIP2A shRNA, respectively (Supplementary Table S1). Additionally, 8 types of phosphorylated proteins with decreased expressions were observed, which were as follows: p53 (S392), MSK1/2 (S376/S360), p53 (S46), AMPK α2 (T172), STAT2 (Y689), STAT5a (Y694), STAT6 (Y641), and PDGF Rβ (Y451) in both cells, of which, the levels of 3 proteins including p53 (S392), MSK1/2 (S376/S360), and STAT5a (Y694) had decreased by over 20% at the same time.

**Fig. 8:**
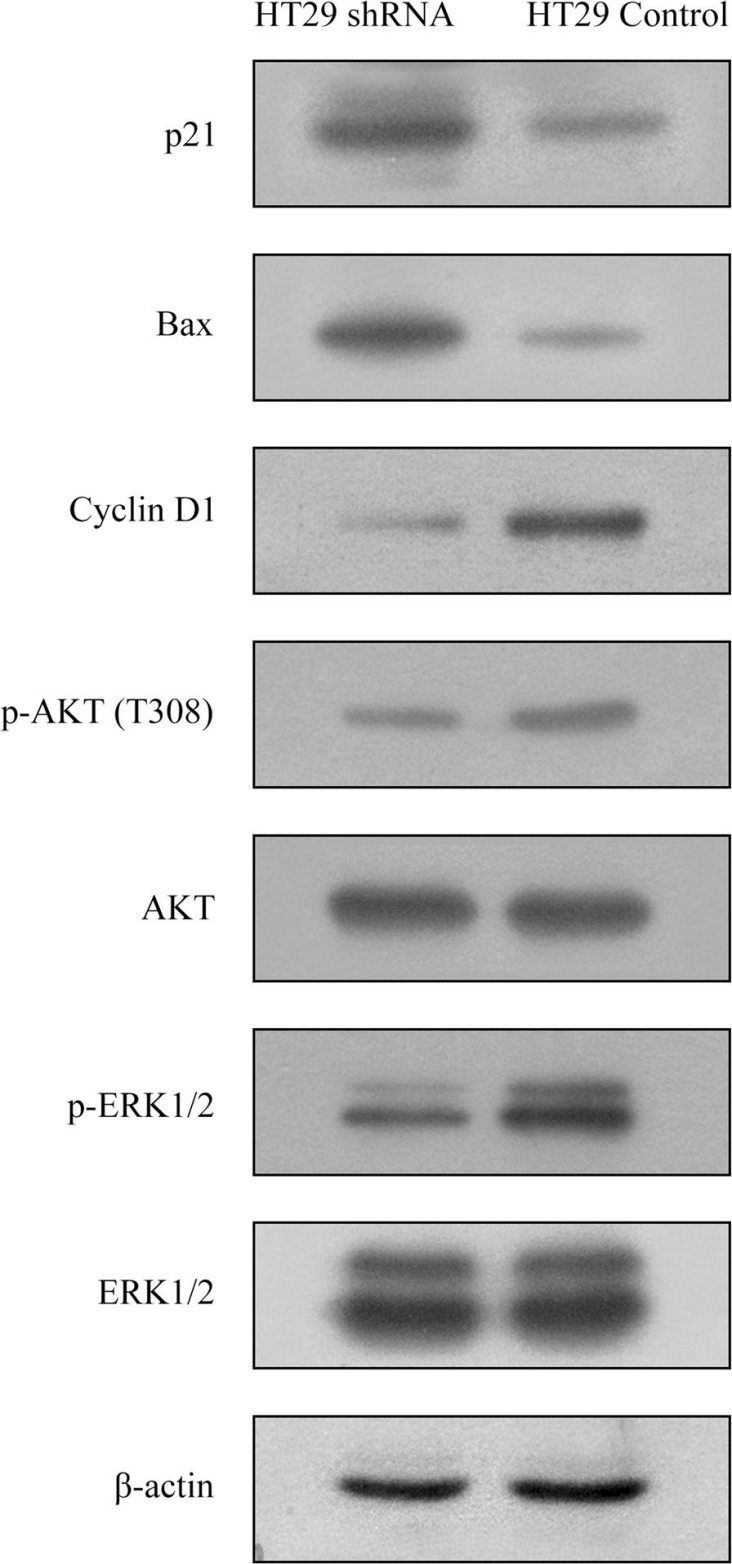
Effect of the CIP2A shRNA on the expression of proteins associated with the cell cycle and apoptosis in HT29 cells. Western blot analysis revealed that the depletion of CIP2A is associated with an increased expression of p21 and Bax, decreased expression of Cyclin D1, and phosphorylation of ERK1/2 and AKT (T308) in HT29 shRNA cells

Based on our experimental results and previously available data in literature, some of these proteins were selected for verification. As shown in Fig. 7c, the differential expression and total levels of p-p53 (S392), p-STAT5a (Y694) could be easily determined. Additionally, we found that the protein levels of Cyclin D1 in CIP2A-shRNA had decreased, while those of p21 and Bax had increased. The level of ERK1/2 and AKT (T308) phosphorylation were significantly inhibited in HT29 cells with CIP2A-shRNA (Fig. 8).

## Discussion

CIP2A is a recently identified biomarker, which can inhibit the activity of PP2A in the malignant tissues of humans. Although we identified that CIP2A acts as an oncoprotein in the CRC tissues compared to normal tissues, using IHC in 2013, the underlying mechanisms of its function in the development of CRC were not entirely clear [15]. In this study, we determined that the mRNA and protein expressions of CIP2A were higher in the CRC tissues than in the corresponding normal tissues. Remarkably, the OS and DFS were better in patients with CRC expressing low levels of the CIP2A protein than those expressing high levels of the protein. Multivariate Cox regression analysis revealed that among the variables analyzed, the expression of CIP2A was an independent prognostic parameter for OS and DFS. This observation was broadly consistent with the data available in literature [16–18]. Based on the data described above, it is probable that determining the expression of CIP2A through IHC could serve as one of the ways for identification of patients with an increased risk of invasion and metastasis of CRC.

In addition to its biological significance in the promotion of the malignant transformation of human cells, CIP2A also plays important roles in the development of cancers. A previous study has demonstrated that CIP2A could regulate the cell cycle by targeting the polo-like kinase (Plk1) and protect cancer cells from apoptosis and senescence. The Plk1 was shown to be an essential role in multiple steps of mitosis [19]. Importantly, the knockdown of CIP2A using siRNAs could inhibit the tumor growth of nasopharyngeal carcinoma in xenografts [20]. Similarly, we also found that silencing the expression of CIP2A suppressed CRC cell proliferation, growth and xenograft tumor growth in vivo. Moreover, the differences in the expressions of VEGF and CD34 between the experimental and control groups were found to be statistically significant (*P* < 0.05). Both proteins are related to the histological grade and biological behavior of CRC cells, which can be used as important reference indices for the prognosis of the disease [21]. It has been suggested that CIP2A may regulate its expression, enhance the biological activity, and further promote the proliferation of vascular endothelial cells and smooth muscle cells and increase the blood supply in tumors, which not only supports the growth of these cells, but also creates favorable conditions for their diffusion and metastasis, the underlying mechanisms of which require further study.

The critical role of C-myc in the development of cancers and its interference with the effects of anticancer drugs has increased the demand for the innovation and application of small molecule inhibitors of C-myc in targeted therapies [22]. The findings of our current study were consistent with those reported in the previously published studies [23–25], in which, 10,058-F4 was found to be effective in inhibiting the proliferation of CRC cells and inducing their apoptosis. Interestingly, expression of the CIP2A protein had decreased with an increase in the concentration of 10,058-F4. Combined with the data of previous studies, in which, CIP2A siRNA had decreased the levels of C-myc [26, 27], the data support the concept that C-myc and CIP2A can stabilize each other by mutual protection from proteolytic degradation at the protein level. Additionally, these results demonstrated that the inhibition of C-myc using either 10,058-F4 or siRNA results in a decrease in the levels of CIP2A in CRC cells. Therefore, further research needs to be conducted on the inhibition of C-myc, particularly because it is a surrogate target of CIP2A.

Compared with the method of histological tumor-specific index detection, serum samples are more convenient to obtain and easy to be popularized and applied in clinical practice, which has better feasibility for long-term follow-up and detection of tumor patients. Although many candidate biomarkers have been identified for this purpose, current biomarker detection assays are not sensitive or specific to provide an early and reliable diagnosis [28–30]. In this regard, efforts to discover new cancer biomarkers still continue. The presence of CIP2A autoantibodies has been detected in the serum of patients with some cancers [31]. However, to date, the presence of anti-CIP2A autoantibodies in patients with CRC has not been reported. In our study, the mean titer of autoantibodies against CIP2A was significantly higher in the sera of patients with CRC than in the sera of normal individuals. Among the large number of tumor markers associated with CRC, CEA and CA19–9 are the ones most commonly used markers in clinical studies [32–34]. It has been proven that methods involving the combined use of several tumor markers are better for diagnosing and monitoring CRC than those employing any single marker. We determined that a significant and positive correlation exists between the levels of CIP2A with those of CEA, CA19–9, and the clinical stages, suggesting that CIP2A plays a role in the development of CRC. However, no significant correlation between CIP2A and the prognosis of CRC in patients was observed. A small sample size could be the problem underlying this observation. Therefore, to further assess if CIP2A could be a specific marker for the diagnosis of CRC, ideally, future projects should involve analysis of a greater number of serum samples to verify.

The mechanisms of CIP2A activation and overexpression in cancer cells have been investigated by several studies, in which some pathways were found to be associated with development of cancer, such as PI3K/mTOR and MAPK/ERK signaling pathway [27, 35]. In the current study, we profiled the phosphokinase signaling pathways associated with CIP2A in different cell lines of CRC and found p-p53 (S392) and p-STAT5a (Y694) to be associated with the depletion of CIP2A expression. According to the data reported in reference studies, the p-p53 (S392) could be associated with an advanced tumor stage [36, 37] and p-STAT5a (Y694) could induce the overexpression of target genes resulting in increased growth and invasion ability of breast cancer cells [38]. Furthermore, based on our results of western blotting combined with those of previous reference studies [16, 39–41], we could identify that the extent of phosphorylation of ERK1/2 and AKT (T308) had decreased in the HT29 shRNA group. These results were different from those of our previous phosphorkinase arrays, which may be due to the differences in our experimental procedures, samples, and reagents. Additionally, some phenomena were found to be consistent with those observed in our former study, in which the expression of Cyclin D1 had clearly been shown to be downregulated [42]. In summary, these results have suggested that CIP2A could regulate cell proliferation and apoptosis via the involvement of multiple pathways and could potentially be used as a drug target for the treatment of CRC.

## Conclusions

In summary, our study describes the expression pattern of CIP2A in CRC tissues and suggests that an elevation in its levels could result in an invasive phenotype and poor prognosis of CRC. Anti-CIP2A autoantibodies could serve as potential biomarkers that could be used to diagnose CRC during clinical serological screening. Additionally, functional and mechanistic studies have indicated that CIP2A plays a key role in the establishment of CRC cells by regulating multiple signaling pathways. Based on the results presented here, we suggest that CIP2A could serve as a novel molecular target for diagnosing CRC.

## Supplementary information

**Additional file 1 Table S1.** The number of phosphorylated proteins decreased in CRC cells with scramble control and CIP2A shRNA.

## Data Availability

Not applicable.

## Abbreviations

CIP2A: Cancerous inhibitor of protein phosphatase 2A;
CRC: Colorectal cancer;
qRT-PCR: Quantitative RT-PCR;
IHC: Immunohistochemistry;
ELISA: Enzyme-linked immunosorbent assay;
siRNA: Small interfering RNA-mediated depletion;
OS: Overall survival;
DFS: Disease-free survival;
HCC: Hepatocellular cancers
*shRNAs*: Short hairpin RNAs

## References

[1] Siegel RL, Miller KD, Jemal A (2018). Cancer statistics, 2018. CA Cancer J Clin.

[2] Deng Y, Chi P, Lan P, Wang L, Chen W, Cui L, Chen D, Cao J, Wei H, Peng X (2019). Neoadjuvant modified FOLFOX6 with or without radiation versus fluorouracil plus radiation for locally advanced rectal Cancer: final results of the Chinese FOWARC trial. J Clin Oncol.

[3] Junttila MR, Puustinen P, Niemela M, Ahola R, Arnold H, Bottzauw T, Ala-aho R, Nielsen C, Ivaska J, Taya Y (2007). CIP2A inhibits PP2A in human malignancies. Cell.

[4] Khanna A, Bockelman C, Hemmes A, Junttila MR, Wiksten JP, Lundin M, Junnila S, Murphy DJ, Evan GI, Haglund C (2009). MYC-dependent regulation and prognostic role of CIP2A in gastric cancer. J Natl Cancer Inst.

[5] Razi Soofiyani S, Mohammad Hoseini A, Mohammadi A, Khaze Shahgoli V, Baradaran B, Hejazi MS (2017). siRNA-Mediated Silencing of CIP2A Enhances Docetaxel Activity Against PC-3 Prostate Cancer Cells. Adv Pharm Bull.

[6] Cristobal I, Zazo S, Torrejon B, Pedregal M, Madoz-Gurpide J, Lluch A, Eroles P, Rovira A, Albanell J, Garcia-Foncillas J, Rojo F (2017). CIP2A confirms its prognostic value in triple-negative breast cancer. Oncogene.

[7] Liu X, Chai Y, Li J, Ren P, Liu M, Dai L, Qian W, Li W, Zhang JY (2014). Autoantibody response to a novel tumor-associated antigen p90/CIP2A in breast cancer immunodiagnosis. Tumour Biol.

[8] He H, Wu G, Li W, Cao Y, Liu Y (2012). CIP2A is highly expressed in hepatocellular carcinoma and predicts poor prognosis. Diagn Mol Pathol.

[9] Huang MJ, Cheng YC, Liu CR, Lin S, Liu HE (2006). A small-molecule c-Myc inhibitor, 10058-F4, induces cell-cycle arrest, apoptosis, and myeloid differentiation of human acute myeloid leukemia. Exp Hematol.

[10] Bashash D, Sayyadi M, Safaroghli-Azar A, Sheikh-Zeineddini N, Riyahi N, Momeny M (2019). Small molecule inhibitor of c-Myc 10058-F4 inhibits proliferation and induces apoptosis in acute leukemia cells, irrespective of PTEN status. Int J Biochem Cell Biol.

[11] Xie Y, Zhang J, Xu Y, Shao C (2012). SirT1 confers hypoxia-induced radioresistance via the modulation of c-Myc stabilization on hepatoma cells. J Radiat Res.

[12] Sampson VB, Rong NH, Han J, Yang Q, Aris V, Soteropoulos P, Petrelli NJ, Dunn SP, Krueger LJ (2007). MicroRNA let-7a down-regulates MYC and reverts MYC-induced growth in Burkitt lymphoma cells. Cancer Res.

[13] Xie D, Sham JS, Zeng WF, Lin HL, Che LH, Wu HX, Wen JM, Fang Y, Hu L, Guan XY (2003). Heterogeneous expression and association of beta-catenin, p16 and c-myc in multistage colorectal tumorigenesis and progression detected by tissue microarray. Int J Cancer.

[14] Chen Y, Zhou Y, Qiu S, Wang K, Liu S, Peng XX, Li J, Tan EM, Zhang JY (2010). Autoantibodies to tumor-associated antigens combined with abnormal alpha-fetoprotein enhance immunodiagnosis of hepatocellular carcinoma. Cancer Lett.

[15] Peng XY, Chen W, Zhou K, Fu JP, Fu P, Zeng QL (2013). Expression of cancerous inhibitor of protein phosphatase 2A in tissue microarray of colorectal cancer and its clinical significance. Zhonghua Wei Chang Wai Ke Za Zhi.

[16] Chen KF, Yen CC, Lin JK, Chen WS, Yang SH, Jiang JK, Lan YT, Lin CC, Yu HC, Hsu HM (2015). Cancerous inhibitor of protein phosphatase 2A (CIP2A) is an independent prognostic marker in wild-type KRAS metastatic colorectal cancer after colorectal liver metastasectomy. BMC Cancer.

[17] Teng HW, Yang SH, Lin JK, Chen WS, Lin TC, Jiang JK, Yen CC, Li AF, Chen PC, Lan YT (2012). CIP2A is a predictor of poor prognosis in colon cancer. J Gastrointest Surg.

[18] Bockelman C, Koskensalo S, Hagstrom J, Lundin M, Ristimaki A, Haglund C (2012). CIP2A overexpression is associated with c-Myc expression in colorectal cancer. Cancer Biol Ther.

[19] Kim JS, Kim EJ, Oh JS, Park IC, Hwang SG (2013). CIP2A modulates cell-cycle progression in human cancer cells by regulating the stability and activity of Plk1. Cancer Res.

[20] Liu N, He QM, Chen JW, Li YQ, Xu YF, Ren XY, Sun Y, Mai HQ, Shao JY, Jia WH (2014). Overexpression of CIP2A is an independent prognostic indicator in nasopharyngeal carcinoma and its depletion suppresses cell proliferation and tumor growth. Mol Cancer.

[21] Bolat F, Kayaselcuk F, Nursal TZ, Yagmurdur MC, Bal N, Demirhan B (2006). Microvessel density, VEGF expression, and tumor-associated macrophages in breast tumors: correlations with prognostic parameters. J Exp Clin Cancer Res.

[22] Dang CV (2012). MYC on the path to cancer. Cell.

[23] Lin CP, Liu JD, Chow JM, Liu CR, Liu HE (2007). Small-molecule c-Myc inhibitor, 10058-F4, inhibits proliferation, downregulates human telomerase reverse transcriptase and enhances chemosensitivity in human hepatocellular carcinoma cells. Anti-Cancer Drugs.

[24] Wang J, Ma X, Jones HM, Chan LL, Song F, Zhang W, Bae-Jump VL, Zhou C (2014). Evaluation of the antitumor effects of c-Myc-max heterodimerization inhibitor 100258-F4 in ovarian cancer cells. J Transl Med.

[25] Kugimiya N, Nishimoto A, Hosoyama T, Ueno K, Enoki T, Li TS, Hamano K (2015). The c-MYC-ABCB5 axis plays a pivotal role in 5-fluorouracil resistance in human colon cancer cells. J Cell Mol Med.

[26] Come C, Laine A, Chanrion M, Edgren H, Mattila E, Liu X, Jonkers J, Ivaska J, Isola J, Darbon JM (2009). CIP2A is associated with human breast cancer aggressivity. Clin Cancer Res.

[27] Wiegering A, Pfann C, Uthe FW, Otto C, Rycak L, Mader U, Gasser M, Waaga-Gasser AM, Eilers M, Germer CT (2013). CIP2A influences survival in colon cancer and is critical for maintaining Myc expression. PLoS One.

[28] Kim ST, Chang WJ, Jin L, Sung JS, Choi YJ, Kim YH (2015). Can serum be used for analyzing the KRAS mutation status in patients with advanced colorectal Cancer?. Cancer Res Treat.

[29] Wang J, Wang X, Lin S, Chen C, Wang C, Ma Q, Jiang B (2013). Identification of kininogen-1 as a serum biomarker for the early detection of advanced colorectal adenoma and colorectal cancer. PLoS One.

[30] Chang W, Wu L, Cao F, Liu Y, Ma L, Wang M, Zhao D, Li P, Zhang Q, Tan X (2011). Development of autoantibody signatures as biomarkers for early detection of colorectal carcinoma. Clin Cancer Res.

[31] Soofiyani SR, Hejazi MS, Baradaran B (2017). The role of CIP2A in cancer: a review and update. Biomed Pharmacother.

[32] Bombski G, Gasiorowska A, Orszulak-Michalak D, Neneman B, Kotynia J, Strzelczyk J, Janiak A, Malecka-Panas E (2003). Elevated plasma gastrin, CEA, and CA 19-9 levels decrease after colorectal cancer resection. Int J Color Dis.

[33] Ning S, Wei W, Li J, Hou B, Zhong J, Xie Y, Liu H, Mo X, Chen J, Zhang L (2018). Clinical significance and diagnostic capacity of serum TK1, CEA, CA 19-9 and CA 72-4 levels in gastric and colorectal cancer patients. J Cancer.

[34] Grotowski M, Maruszynski M, Piechota W (2001). Usefulness of preoperative assay CEA and CA 19-9 in colorectal cancer patients. Pol Merkur Lekarski.

[35] Bartalucci N, Calabresi L, Balliu M, Martinelli S, Rossi MC, Villeval JL, Annunziato F, Guglielmelli P, Vannucchi AM (2017). Inhibitors of the PI3K/mTOR pathway prevent STAT5 phosphorylation in JAK2V617F mutated cells through PP2A/CIP2A axis. Oncotarget.

[36] Bar JK, Slomska I, Rabczynki J, Noga L, Grybos M (2009). Expression of p53 protein phosphorylated at serine 20 and serine 392 in malignant and benign ovarian neoplasms. Correlation with clinicopathological parameters of tumors. Int J Gynecol Cancer.

[37] Yap DB, Hsieh JK, Zhong S, Heath V, Gusterson B, Crook T, Lu X (2004). Ser392 phosphorylation regulates the oncogenic function of mutant p53. Cancer Res.

[38] Dho SH, Kim JY, Lee KP, Kwon ES, Lim JC, Kim CJ, Jeong D, Kwon KS (2016). STAT5A-mediated NOX5-L expression promotes the proliferation and metastasis of breast cancer cells. Exp Cell Res.

[39] Zhang X, Xu B, Sun C, Wang L, Miao X (2015). Knockdown of CIP2A sensitizes ovarian cancer cells to cisplatin: an *in vitro* study. Int J Clin Exp Med.

[40] Cristobal I, Manso R, Rincon R, Carames C, Senin C, Borrero A, Martinez-Useros J, Rodriguez M, Zazo S, Aguilera O (2014). PP2A inhibition is a common event in colorectal cancer and its restoration using FTY720 shows promising therapeutic potential. Mol Cancer Ther.

[41] Liu X, Peng B, Li Y, Lei N, Li W, Zhang JY (2014). p90/CIP2A mediates breast cancer cell proliferation and apoptosis. Mol Biol Rep.

[42] Fang Y, Li Z, Wang X, Zhang S (2012). CIP2A is overexpressed in human ovarian cancer and regulates cell proliferation and apoptosis. Tumour Biol.

