# Supplementary material for: Effect of CIP2A and its mechanism of action in the malignant biological behavior of colorectal cancer
Source: Cell Commun Signal. 2020 Apr 22;18:67. doi: 10.1186/s12964-020-00545-6 (PMC7178757; doi:10.1186/s12964-020-00545-6)
Supplement: Supplementary file 2 — Additional file 1 Table S1. The number of phosphorylated proteins decreased in CRC cells with scramble control and CIP2A shRNA. [file 12964_2020_545_MOESM2_ESM.doc]

**Table S1. The number of phosphorylated proteins decreased in CRC cells with scramble control and CIP2A shRNA**

|  | HT29 | DLD1 |
| --- | --- | --- |
| Decrease in the phosphorylation levels | p53 (S392), MSK1/2 (S376/S360), AMPKα2 (T172), c-Jun (S63), Src (Y419), STAT2 (Y689), STAT5a (Y694), RSK1/2/3 (S380/S386/S377), Fyn (Y420), STAT6 (Y641), p27 (T198), PLC-γ1 (Y783), PDGF Rβ (Y451), STAT3 (S727), WNK1 (T60), PRAS40 (T246), p53 (S46) | ERK1/2 (T202/Y204,T185/Y187), JNK pan (T183/Y185,T221/Y223), GSK-3β (S21/S9), p53 (S392), MSK1/2 (S376/S360), AMPKα1 (T174), Akt (S473), Akt (T308), p53 (S46), CREB (S133), HSP27 (S78/S82), AMPKα2 (T172), β-Catenin, p70 S6 Kinase (T389), Lyn (Y397), Lck (Y394), STAT2 (Y689), STAT5a (Y694), Yes (Y426), Fgr (Y412), STAT6 (Y641), STAT5b (Y699), STAT3 (Y705), Chk-2 (T68), PDGF Rβ (Y451), HSP60 |
| Decrease in the phosphorylation levels over 20% | p53 (S392), MSK1/2 (S376/S360), AMPKα2 (T172), STAT5a (Y694), RSK1/2/3 (S380/S386/S377), p27 (T198), PLC-γ1 (Y783), PRAS40 (T246) | ERK1/2 (T202/Y204,T185/Y187), JNK pan (T183/Y185,T221/Y223), GSK-3β (S21/S9), p53 (S392), MSK1/2 (S376/S360), AMPKα1 (T174), Akt (S473), Akt (T308), p53 (S46), CREB (S133), β-Catenin, p70 S6 Kinase (T389), Lyn (Y397), Lck (Y394), STAT5a (Y694), Fgr (Y412), STAT6 (Y641), STAT5b (Y699), STAT3 (Y705), Chk-2 (T68), PDGF Rβ (Y451), HSP60 |
